# Relationship Between Weight Loss, Changes in Serum hs-CRP Levels and apo A-1 Lipoprotein, and High-Density Lipoprotein-Cholesterol Ratios as Predictors for Improved Cardiovascular Risk Factors After Laparoscopic Sleeve Gastrectomy

**DOI:** 10.1007/s11695-024-07441-9

**Published:** 2024-08-14

**Authors:** Mohamed Hany, Hala M. Demerdash, Anwar Ashraf Abouelnasr, Ann Samy Shafiq Agayby, Mohamed Ibrahim, Ramy E. Arida, Bart Torensma

**Affiliations:** 1 Department of Surgery, Medical Research Institute, Alexandria University, 165 Horreya Avenue, Hadara, 21561 Alexandria Egypt; 2 Madina Women’s Hospital, Alexandria, Egypt; 3 Clinical Pathology, Alexandria University, Alexandria, Egypt; 4 Alexandria Faculty of Medicine, Alexandria, Egypt; 5 Leiden University Medical Center (LUMC), Leiden, The Netherlands

**Keywords:** Dyslipidemia, Total cholesterol, High-density lipoprotein-cholesterol insulin resistance, Cardiovascular risk, Apo A-1 lipoprotein

## Abstract

**Introduction:**

Obesity, a major global health concern, is a known risk factor for cardiovascular disease (CVD), often due to dyslipidemia and insulin resistance. Laparoscopic sleeve gastrectomy (LSG) is an effective weight reduction surgery that not only alters body metabolism and gastrointestinal physiology but also significantly lowers cardiovascular disease risk.

**Methods:**

This study explores the impact of weight loss on serum high-sensitivity C-reactive protein (hs-CRP), an established inflammatory marker, and changes in cardiovascular risk factors, particularly high-density lipoprotein-cholesterol (HDL-C) ratios, serum apo A-1, lipid profile, and HOMA-IR in severe obesity undergoing LSG. Anthropometric measurements and blood samples were collected preoperatively and 6 months postoperatively to hs-CRP, HOMA-IR, lipid profile, apo A-1, and low- and high-density lipoprotein-cholesterol (LDL-C/HDL-C) ratios, total cholesterol to HDL-C (TC/HDL-C) ratio, and monocyte to high-density lipoprotein-cholesterol ratio (MHR).

**Results:**

In total, 70 patients were analyzed after 6 months and reached %TWL 27.4 ± 9.5 and %EWL 62.0 ± 15.4. Significant improvements were noted in all measured biomarkers. Analysis showed that each unit reduction in BMI significantly affected hs-CRP and HDL-C. Furthermore, moderate associations between hs-CRP and various cardiovascular disease risk biomarkers, including a negative correlation with apo A-1 and positive correlations with total cholesterol (TC), TC/HDL-C, and LDL-C/HDL-C, along with a mild positive correlation with HOMA-IR.

**Conclusion:**

Weight loss following LSG significantly reduced inflammation and improved atheroprotection. Improved inflammation markers were associated with favorable changes in cardiovascular risk factors, including HDL-C ratios particularly TC/HDL-C, LDL-C/HDL-C, and apo A-1.

**Graphical Abstract:**

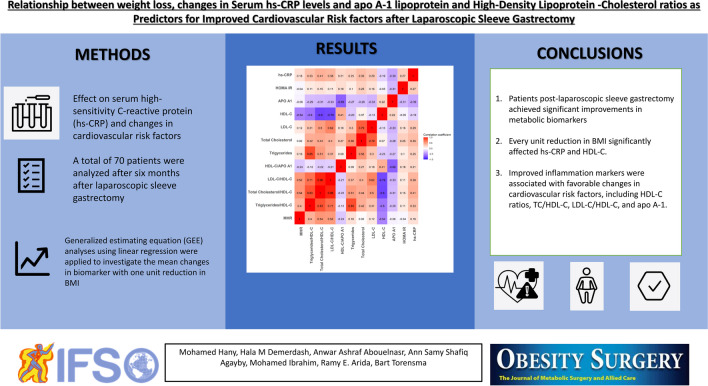

## Introduction

The obesity epidemic poses a significant public health challenge, primarily due to its role in fostering associated medical problems, including cardiovascular disease (CVD) [1, 2]. This condition is characterized by excessive fat accumulation and dysfunctional adipose tissue, potentially leading to a chronic inflammatory state detrimental to overall health. A key consequence of obesity is an increased CVD risk, driven by dyslipidemia, insulin resistance, and hypertension, with the most common cardiovascular risk factors including high blood pressure, elevated cholesterol levels, smoking, diabetes, obesity, physical inactivity, unhealthy diet, family history of heart disease, age, and chronic stress [3]. Dyslipidemia involves abnormal lipid levels in the blood, characterized by elevated triglycerides (TG), high low-density lipoprotein-cholesterol (LDL-C), and reduced high-density lipoprotein-cholesterol (HDL-C) [4]. HDL-C, known as “good cholesterol,” is crucial for lipid biodistribution via reverse cholesterol transport (RCT), facilitating cholesterol removal from tissues and preventing its deposition in blood vessels [5]. It also possesses anti-atherogenic and anti-inflammatory properties, contributing significantly to endothelial function maintenance [5, 6]. The monocyte to high-density lipoprotein-cholesterol ratio (MHR) is an emerging biomarker for cardiovascular risk, combining inflammatory markers (monocytes) with lipid metabolism indicators (HDL cholesterol). Elevated monocyte levels signify increased inflammation, while high HDL cholesterol levels are protective against cardiovascular disease. A higher MHR value reflects a greater inflammatory state and lower osteoprotective capacity, making it a valuable tool for assessing cardiovascular risk in various patient populations [7]. Apolipoprotein A-I (apo A-1) is essential in lipid metabolism and cardiovascular protection. As the primary component of HDL-C, it facilitates reverse cholesterol transport, which is crucial for preventing arterial cholesterol accumulation and reducing atherosclerosis risk. Additionally, apo A-1 has anti-inflammatory and antioxidant properties, playing a role in preventing LDL-C oxidation and improving endothelial function. These functions highlight apo A-1 as a key factor in managing dyslipidemia and preventing cardiovascular disease [8, 9].

Recent studies challenge the traditional view that high HDL-C levels invariably correlate with reduced CVD risk. This suggests that HDL-C functionality might be a more accurate indicator of cardiovascular health through its anti-inflammatory and antioxidant properties [10, 11]. HDL-C functionality might be a more accurate indicator of cardiovascular health due to its anti-inflammatory and antioxidant properties. HDL-C plays a crucial role in reverse cholesterol transport, helping to remove excess cholesterol from arterial walls and transport it to the liver for excretion. Additionally, HDL-C has anti-inflammatory properties that can reduce inflammation in the cardiovascular system and antioxidant properties that help protect against oxidative damage to lipids and proteins. These functions collectively contribute to maintaining cardiovascular health and reducing the risk of cardiovascular disease.

This shift in understanding has sparked interest in the HDL-C/apo A-I ratio as a potential atherosclerosis predictor [12]. Additionally, metabolic bariatric surgery (MBS) has shown promise in improving HDL-C levels, though the impact of MBS on HDL function is still less characterized [8]. Furthermore, CRP belongs to the pentraxin family of proteins, an acute-phase reactant protein [13], and exhibits dual roles in this context. Primarily produced by the liver, CRP is also synthesized in patients with obesity in response to inflammatory mediators, mainly interleukin-6 (IL-6) [14]. Compared to other acute-phase reactants, CRP levels are relatively stable, allowing for accurate estimation [14, 15], highlighting the multifaceted role of high-sensitivity CRP (hs-CRP), not just as an inflammatory biomarker but also as an active participant in atherogenesis. It suppresses endothelial nitric oxide synthase (eNOS) activity and inhibits nitric oxide (NO) production, which can activate nuclear factor kappa β and trigger the release of adhesion molecules [16, 17]. Understanding the dynamics of hs-CRP is crucial in assessing CVD presence and monitoring the health status of patients with obesity.

Accordingly, high-sensitivity C-reactive protein (hs-CRP) is a predictive inflammatory biomarker regarded as a crucial indicator of atherosclerosis development. It is widely employed in clinical practice as a risk marker for CVD. The relationship between weight loss and serum hs-CRP levels, a recognized inflammatory biomarker, is studied in patients undergoing laparoscopic sleeve gastrectomy (LSG) [13, 18]. The objective of this study was to investigate changes in cardiovascular risk factors, including serum hs-CRP, serum apo A-1, various HDL-C ratios, lipid profiles, and the Homeostatic Model Assessment for Insulin Resistance (HOMA-IR) 6 months after MBS, and to determine the predictive value of HDL-C ratios and apo A-1 for improved cardiovascular risk after weight loss.

## Material and Methods

This prospective cohort study enrolled patients with obesity before and after LSG procedure to test cardiovascular risk factors. It was conducted at Medical Research Institute, Alexandria University Hospitals, and Madina Women’s Hospital, Alexandria, Egypt, between January 2023 and April 2023 with 6 months of follow-up. The study was conducted in accordance with the principles of the Declaration of Helsinki and approved by the ethical committee board. All patients signed informed consent.

### *Study Endpoints*

**The primary endpoint** is to validate the clinical utility of weight loss and serum hs-CRP levels as established inflammatory biomarkers and CV risk factors in patients undergoing LSG.

**The secondary endpoint** is to evaluate the impact of changes from serum apo A-1 as well as HDL-C ratios [HDL-C/apo A-1, LDL-C/HDL-C, total cholesterol (TC)/HDL-C, TG/HDL-C, and MHR ratios], lipid profile, and HOMA-IR in patients undergoing LSG.

### Inclusion Criteria

Patients eligible for metabolic bariatric surgery (MBS) were those aged between 18 and 60 years, presenting with a body mass index (BMI) greater than 35 kg/m^2^, either with or without associated medical problems [19].

### Exclusion Criteria

(1) Patients who had previously undergone MBS, (2) traditional CV risk factors (such as smoking, family history of premature coronary artery disease, or receiving medications (beta-blockers or steroids)), (3) patients with liver disease, and (4) patients with endocrine disorder as thyroid.

### Data Collection

All data and tests were performed, obtained, and compared preoperative up to 6 months postoperatively.

Preoperative and postoperative: Baseline characteristics (age, sex, body mass index (BMI)), associated medical problems, lab results, biomarkers, and weight loss (WL) assessed by the percentage of total WL (%TWL) and excess WL (%EWL).

Hypertension was defined as receiving antihypertension treatment or systolic blood pressure (BP) above 140 mmHg or diastolic BP above 90 mmHg. Insulin resistance (IR) is clinically defined as a subnormal biological response to either exogenously administered or endogenously secreted insulin. Diagnosis of insulin resistance was defined using The Homeostasis Model Assessment of Insulin Resistance (HOMA-IR) with values above 2 or hemoglobin A1c levels greater than 6.5% at baseline [20].

Diagnosis of dyslipidemia was confirmed by the presence of one or more disorders in lipid parameters TC > 200 mg/dl; TG > 150 mg/dl; LDL-C > 135 mg/dl; and/or HDL-C < 35 mg/dl in men or < 39 mg/dl in women or receiving lipid-lowering drugs [21].

### Anthropometric Measurements

Height was measured with high precision to the nearest 0.1 cm. Weight was determined in lightweight clothing and measured to the nearest 0.1 kg. BMI was calculated as weight in kilograms divided by the square of height in meters. Weight loss was calculated as the percentage of excess BMI loss (100 × [baseline BMI − postoperative BMI] / [baseline BMI − 25]) and percentage of excess weight loss (%EWL = (initial body weight − postoperative body weight) / (initial body weight − ideal body weight for BMI 25) × 100%). Total weight loss percentage (%TWL = (initial body weight − postoperative body weight) / initial body weight) was also calculated.

### Laboratory Measurements

Laboratory assessments were conducted using standard methods on fasting venous blood samples taken between 8:00 a.m. and 9:00 a.m. after a 12-h overnight fast. Collected blood samples were divided into two tubes: 1 cm^3^ was taken into a vacuum-sealed, lavender-capped tube for complete blood count (CBC), and 5 cm^3^ was delivered into a vacuum-sealed, yellow-capped serum tube for biochemical tests. Serum tubes were centrifuged at 4000 rpm for 10 min within half an hour, and the serums were separated. Hemolyzed serum samples were excluded.

Routine biochemistry measurements, including serum glucose, total cholesterol (TC), HDL-C, and triglycerides (TG), were measured on a Hitachi 7180 Biochemistry Automatic Analyzer (Hitachi, Japan). LDL-C was calculated using Friedewald’s formula: (TC)—(HDL-C)—(TG/5). A full workup of all lab tests is provided in the Appendix. Serum hs-CRP measurements were performed immediately. Serum for other measurements, including apo A-1 and serum insulin, was stored at − 80 °C until the day of analysis.

### Surgical Procedure

Standard 5 ports were used: three 12-mm ports (for the camera, right and left working ports) and two 5-mm ports (for liver retraction and the assistant). Pneumo-peritoneum was created after using optical trocars for entry. The greater omentum was dissected off the greater curvature of the stomach using the EnSeal device (Ethicon Endo-Surgery, Cincinnati, OH, USA), followed by dissection of any posterior gastric adhesions and excision of Belsey’s pad of fat. The gastric sleeve was created over a 40-Fr calibration bougie using an Echelon Flex Endopath 60-mm linear stapler (Ethicon Endo-Surgery, Cincinnati, OH, USA) for gastric division, starting at 3–5 cm before the pylorus, up to the angle of His using green, gold, and blue reloads according to the thickness of tissues. The staple line was invaginated completely by running seromuscular stitches using unidirectional absorbable 3/0 V-Loc 180 sutures (Covidien, Mansfield, MA, USA).

### Statistical Analysis

Descriptive and inferential statistics were used for the analyses. All data were tested for normality using the Kolmogorov–Smirnov, Q-Q plot, and Levene’s tests. Categorical variables are expressed as numbers and percentages. Normally and non-normally distributed continuous variables are presented as means with standard deviations (SDs) and medians with interquartile ranges. When appropriate, categorical variables were tested using Pearson’s chi-square or Fisher’s exact test. Normally distributed continuous data were tested with dependent samples with Student’s *t*-test, or the Wilcoxon signed-rank test was used for skewed (nonparametric) data. Generalized estimating equation (GEE) analyses using linear regression were applied to investigate the mean changes in biomarker with one unit reduction in BMI. Correlations of the cardiovascular biomarkers with hs-CRP were estimated by Pearson’s correlation coefficients if normal distributed or Spearman’s rank test if not normally distributed. A significant level of 0.05 was used in all analyses. All analyses were conducted using R software version 4.2.2.

### Sample Size

The sample size calculation was done using the “pwr” package version 1.3–0. A medium effect size (Cohen’s *D*) of 0.5 for the change in inflammatory biomarkers using paired *t* test and a power of 80% with a significance level of 0.05 was used; this resulted in a minimum sample size of 34 patients. A total of 78 patients were recruited.

## Results

### Baseline Characteristics

This study included 78 patients who underwent an LSG operation in 2023. In total, 8 patients were lost to follow-up (− 8.9%). The final analysis included 70 patients. The mean age was 37.9 ± 12.7 years. 72.9% were female with a preoperative BMI of 45.8 ± 8.1. The most common associated medical problems were 50% apnea, 37.1% diabetes, 30% osteoarthritis, and 20% hypertension (Table 1).

**Table 1:** Baseline characteristics

Characteristics	*N* = 70
Age (years), mean ± standard deviation	37.9 ± 12.7
Sex (female), *n* (%)	51 (72.9%)
Anthropometrics	
Height (M), mean ± standard deviation	1.7 ± 0.1
Weight (kg), mean ± standard deviation	126.3 ± 22.7
BMI (kg/m^2^), mean ± standard deviation	45.8 ± 8.1
Associated medical problems, *n* (%)	
Apnea	35 (50.0%)
Diabetes	26 (37.1%)
Osteoarthritis	21 (30.0%)
Hypertension	14 (20.0%)
Hypothyroidism	7 (10.0%)
Insulin resistance	5 (7.1%)
COPD	5 (7.1%)
Dyslipidemia	4 (5.7%)
GERD	4 (5.7%)
Thrombosis	4 (5.7%)
PCOS	4 (5.7%)

### Weight Loss

Weight loss after 6 months was for %TWL 27.4 ± 9.5 and %EWL 62.0 ± 15.4, corresponding with a mean BMI of 32.7 ± 3.4 (*p* =  < 0.001) (Table 2).

**Table 2:** Weight loss outcomes after operation

Variable	Before	After	*p*
Weight (kg), mean ± standard deviation	126.3 ± 22.7	90.1 ± 11.2	< .001*
BMI (kg/m^2^), mean ± standard deviation	45.8 ± 8.1	32.7 ± 3.4	< .001*
%EWL, mean ± standard deviation	62.0 ± 15.4		
%TWL, mean ± standard deviation	27.4 ± 9.5		

### Changes in Predictive Cardiovascular Risk Biomarkers 6 Months After LSG

The mean levels of hs-CRP decreased, with a mean change of − 2.72 (95% CI: − 3.20 to − 2.24, *p* =  < 0.001). Similarly, HOMA-IR significantly decreased with a mean change of − 1.61 (95% CI: − 2.31 to − 0.91, *p* =  < 0.001), indicating enhanced insulin sensitivity (Table 3).

**Table 3:** GEE analyses of the changes in the levels of predictive cardiovascular risk biomarkers 6 months after LSG

Variable	Before	After	Mean change after LSG	*p*
	Mean ± SD	Mean ± SD	Est (95% CI)	
hs-CRP (mg/l)	4.6 ± 1.9	1.9 ± 0.7	− 2.72 (− 3.20, − 2.24)	< .001*
HOMA-IR	3.2 ± 2.7	1.6 ± 1.3	− 1.61 (− 2.31, − 0.91)	< .001*
Lipid profile				
Apo A-1 (mg/dl)	110.5 ± 30.9	148.1 ± 29.2	37.58 (27.70, 47.47)	< .001*
HDL-C (mg/dl)	41.0 ± 11.2	46.6 ± 7.0	5.51 (2.43, 8.59)	< .001*
LDL-C (mg/dl)	136.8 ± 23.8	113.5 ± 16.6	− 23.22 (− 29.97, − 16.46)	< .001*
TC (mg/dl)	210.8 ± 28.1	186.3 ± 18.9	− 24.49 (− 32.36, − 16.62)	< .001*
TG (mg/dl)	175.1 ± 69.4	133.8 ± 41.8	− 41.31 (− 60.17, − 22.46)	< .001*
HDL ratios				
HDL-C/apo A-1	0.4 ± 0.2	0.3 ± 0.1	− 0.07 (− 0.12, − 0.03)	. 001*
LDL-C/HDL-C	3.6 ± 1.2	2.5 ± 0.6	− 1.10 (− 1.42, − 0.78)	< .001*
TC/HDL-C	5.5 ± 1.7	4.1 ± 0.7	− 1.43 (− 1.86, − 1.00)	< .001*
TG/HDL-C	4.7 ± 2.8	3.0 ± 1.1	− 1.74 (− 2.45, − 1.04)	< .001*
MHR	15.9 ± 7.4	13.7 ± 3.8	− 2.19 (− 4.12, − 0.26)	.034*

^*^Statistically significant (*p* < .05)

### Lipid Profile

Within the lipid profile, significant changes were observed postoperatively at 6 months. Apo A-1 and HDL-C levels increased, with mean changes of 37.58 (95% CI: 27.70 to 47.47, *p* ≤ 0.001) and 5.51 (95% CI: 2.43 to 8.59, *p* ≤ 0.001), respectively. Conversely, LDL-C, TC, and TG levels demonstrated a notable decrease, with mean changes of − 23.22 (95% CI: − 29.97 to − 16.46, *p* ≤ 0.001), − 24.49 (95% CI: − 32.36 to − 16.62, *p* ≤ 0.001), and − 41.31 (95% CI: − 60.17 to − 22.46, *p* ≤ 0.001).

Furthermore, HDL ratios showed a significant reduction, indicative of a positive shift in lipid profiles and cardiovascular health. The HDL-C/apo A-1 ratio decreased by a mean of − 0.07 (95% CI: − 0.12 to − 0.03, *p* = 0.001). Similarly, the LDL-C/HDL-C and TC/HDL-C ratios decreased with mean changes of − 1.10 (95% CI: − 1.42 to − 0.78, *p* ≤ 0.001) and − 1.43 (95% CI: − 1.86 to − 1.00, *p* ≤ 0.001), respectively. The TG/HDL-C ratio was also significantly reduced, with a mean change of − 1.74 (95% CI: − 2.45 to − 1.04, *p* ≤ 0.001). Finally, the monocyte to HDL-C ratio (MHR) exhibited a noteworthy decrease, with a mean change of − 2.19 (95% CI: − 4.12 to − 0.26, *p* = 0.034) (Table 3).

### Changes in Biomarkers Associated with One Unit Reduction in BMI

Every one-unit reduction in BMI had a significant effect on the levels of hs-CRP, with an estimated reduction of 0.10 (95% CI: 0.05 to 0.15, *p* =  < 0.001) and HDL-C levels with an estimated increase of -0.41(95% CI: (-0.83 to -0.01, *p* = 0.05) (Table 4).

**Table 4:** Linear regression analyses for the effect of one unit reduction in the BMI on the levels of predictive cardiovascular risk biomarkers

Covariate	Estimate	95% CI	*p*
hs-CRP (mg/l) reduction	0.10	(0.05, 0.15)	< .001*
HOMA-IR reduction	0.07	(− 0.01, 0.15)	.098
Lipid profile			
Apo A-1 (mg/dl) increase	− 0.43	(− 1.64, 0.78)	.492
HDL-C (mg/dl) increase	− 0.41	(− 0.83, 0.01)	.050*
LDL-C(mg/dl) reduction	− 0.37	(− 1.29, 0.54)	.427
TC (mg/dl) reduction	0.24	(− 0.76, 1.23)	.641
TG (mg/dl) reduction	0.14	(− 2.26, 2.55)	.906
H DL ratios			
HDL-C/apo A-1 reduction	0.00	(0.00, 0.01)	.457
LDL-C/HDL-C reduction	− 0.02	(− 0.06, 0.02)	.272
TC/HDL-C reduction	− 0.02	(− 0.07, 0.03)	.474
TG/HDL-C reduction	− 0.01	(− 0.10, 0.08)	.832
MHR reduction	− 0.07	(− 0.35, 0.22)	.651

^*^Statistically significant (*p* < .05)

### Associations of the Cardiovascular Risk Biomarkers with hs-CRP

HOMA-IR showed a significant positive association, with a mean change in hs-CRP of 0.24 (95% CI: 0.09 to 0.39, *p* = 0.001).

### Lipid Profile

Apo A-1 exhibited a significant mean decrease in high-sensitivity C-reactive protein (hs-CRP) levels of − 0.02 (95% CI: − 0.03 to − 0.01, *p* < 0.001). Significant associations between hs-CRP levels and other lipid parameters were also observed: HDL-C (− 0.04, 95% CI: − 0.08 to − 0.01, *p* = 0.05), LDL-C (0.02, 95% CI: 0.01 to 0.04, *p* = 0.004), TC (0.03, 95% CI: 0.01 to 0.04, *p* =  < 0.001), and TG (0.01, 95% CI: 0.00 to 0.01, *p* = 0.005).

Regarding HDL ratios, the HDL-C/apo A-1, LDL-C/HDL-C, TC/HDL-C, and TG/HDL-C ratios all showed significant correlations with hs-CRP, with mean changes in hs-CRP of 2.94 (95% CI: 0.53 to 5.35, *p* = 0.017), 0.68 (95% CI: 0.35 to 1.01, *p* =  < 0.001), 0.55 (95% CI: 0.33 to 0.76, *p* =  < 0.001), and 0.29 (95% CI: 0.16 to 0.42, *p* =  < 0.001), respectively. Lastly, the monocyte to HDL-C ratio (MHR) did not demonstrate a significant association (Table 5).

**Table 5:** GEE-based analyses the changes in the levels of hs-CRP associated with unit increases of the other predictive cardiovascular risk biomarkers

Covariate	Mean change in hs-CRP	95% CI	*p*
HOMA-IR	0.24	(0.09, 0.39)	.001*
Lipid profile			
Apo A-1 (mg/dl)	− 0.02	(− 0.03, − 0.01)	< .001*
HDL-C (mg/dl)	− 0.04	(− 0.08, 0.01)	.05*
LDL-C (mg/dl)	0.02	(0.01, 0.04)	.004*
TC (mg/dl)	0.03	(0.01, 0.04)	< .001*
TG (mg/dl)	0.01	(0.00, 0.01)	.005*
HDL ratios			
HDL-C/apo A-1	2.94	(0.53, 5.35)	.017*
LDL-C/HDL-C	0.68	(0.35, 1.01)	< .001*
TC/HDL-C	0.55	(0.33, 0.76)	< .001*
TG/HDL-C	0.29	(0.16, 0.42)	< .001*
MHR	0.05	(− 0.01, 0.12)	.093

^*^Statistically significant (*p* < .05)

### Correlation Coefficients Matrix

**Fig. 1:**
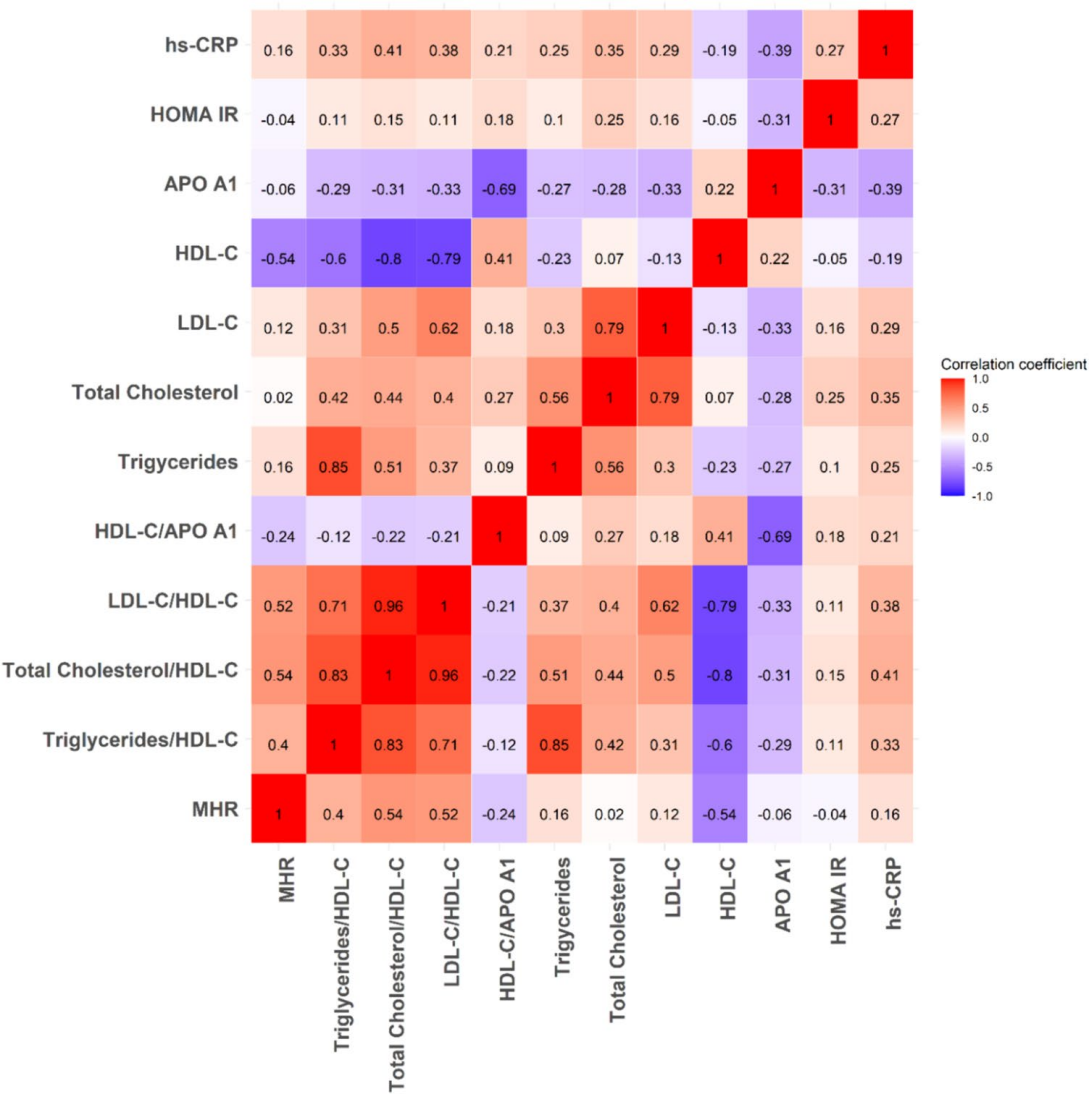
Correlation coefficients of various cardiovascular risk biomarkers with serum hs-CRP

The correlation coefficients of various cardiovascular biomarkers with high-sensitivity C-reactive protein (hs-CRP) were tested and arranged from the strongest to the weakest correlation. The analysis showed that the TC/HDL-C ratio had the most pronounced positive correlation with hs-CRP, evidenced by a moderate correlation coefficient of 0.41 (*p* =  < 0.001). Close behind, apo A-1 displayed a moderate negative correlation with hs-CRP, with a coefficient of − 0.39 (*p* =  < 0.001). Furthermore, the LDL-C/HDL-C ratio, TC, and TG/HDL-C also showed moderate positive correlations, with coefficients of 0.38, 0.35, and 0.33, respectively (*p* =  < 0.001) (Table 6, Fig. 1).

**Table 6:** Correlation coefficients of the cardiovascular risk biomarkers with hs-CRP

Biomarker	Correlation with hs-CRP	*p*
TC/HDL-C	0.41	< .001*
Apo A-1 (mg/dl)	-0.39	< .001*
LDL-C/HDL-C	0.38	< .001*
TC (mg/dl)	0.35	< .001*
TG/HDL-C	0.33	< .001*
LDL-C (mg/dl)	0.29	< .001*
HOMA-IR	0.27	.001*
TG (mg/dl)	0.25	.003*
HDL-C/apo A-1	0.21	.014*
HDL-C (mg/dl)	-0.19	.022*
MHR	0.16	.059

## Discussion

This prospective study was designed to examine the relationship between weight loss and changes in high-sensitivity C-reactive protein (hs-CRP) levels, an established inflammatory biomarker, alongside CV risk factors in patients undergoing LSG. The findings revealed substantial postoperative reductions in BMI and both percentage of %EWL and %TWL. Moreover, notable improvements were observed in hs-CRP, HOMA-IR, serum apo A-1, and lipid parameters, particularly HDL-C ratios. These results indicate a significant correlation between reduced BMI, diminished inflammation, and improved cardiovascular health markers post-surgery. Abnormal metabolites like inflammatory cytokines, free fatty acids, and lipids in adipose tissue drive systemic inflammation in obesity [22]. This inflammation and endothelial dysfunction initiate the early stages of atherosclerosis and contribute to the pathophysiological changes leading to CVD [23].

Weight loss is a fundamental strategy for reducing CV disease risk in patients with obesity, as evidenced by numerous studies [24]. MBS, in particular, not only induces weight loss but also leads to significant improvements in metabolic parameters and insulin resistance in cases of severe obesity [24]. Consequently, body weight reduction following MBS is associated with a marked decrease in circulating levels of hs-CRP, attributable to the reduction in total body fat, including both subcutaneous and visceral fat. This reduction directly impacts CVD risk [18, 25]. In our study, we observed that HDL-C levels were significantly altered per unit reduction in BMI. Supporting this observation, Heffron et al.’s research established a clear connection between low HDL-C levels and elevated BMI and adiposity. Their findings indicate that MBS leads to enhancements in both HDL structure and function [26]. The relationship between HDL-C and BMI may be explained by changes in adipose tissue lipoprotein lipase enzyme activity, decreased hepatic lipase activity, and reduced serum levels of free fatty acid [26]. Additionally, Zvintzou et al. already provided insight into how reducing obesity-associated systemic inflammation through MBS could improve HDL functionality. Their animal model study revealed that postoperative increases in peroxisome proliferator–activated receptor alpha (PPARα) expression stimulate apo A-1 production, which is crucial for enhancing HDL function [27]. Other research has indicated that the resolution of associated medical problems post-MBS is not solely due to weight loss. Instead, it involves factors independent of weight, such as alterations in gut hormones, bile acids, the nervous system, and changes in gut microbiota, as well as the improvement of chronic inflammation [28, 29].

Moreover, our study found that changes in hs-CRP levels correlated with other predictive CV biomarkers. Specifically, there was a mild positive association with the HOMA-IR, with a correlation coefficient of *r* = 0.27 (*p* = 0.001). This suggests that increased hs-CRP levels may exacerbate HOMA-IR by affecting insulin signaling. This is achieved through phosphorylation of the IRS-1, which interferes with insulin-stimulated glucose uptake by impairing the translocation of the GLUT4, thereby contributing to IR [30, 31]. In line with this, Sachan et al. demonstrated that MBS not only affects the pro-inflammatory state but also leads to a significant improvement in HOMA-IR [32].

Improved lipid parameters in our study were associated with reductions in hs-CRP. This association suggests that the postoperative reduction in chronic inflammation is linked with decreased adipogenesis in peripheral subcutaneous adipose tissue, potentially ameliorating adipose tissue dysfunction and metabolic diseases [33]. This was observed as a reduction in TC improvements in reverse cholesterol transport (RCT) with lowered LDL-C, decreased TG, and enhanced levels of HDL-C and apo A-1 [33].

Our findings indicate a negative association between apo A-1 and hs-CRP, suggesting an inverse relationship between apo A-1 levels and systemic inflammation. The observed postoperative increase in apo A-1 is indicative of elevated levels of circulating anti-atherogenic lipoproteins. Beyond its role in inhibiting lipopolysaccharide (LPS)–induced inflammation, neutrophil suppression, and monocyte-macrophage recruitment, apo A-1 has broader implications in cardiovascular health. Apo A-1 contributes to cholesterol efflux and reverse cholesterol transport, processes fundamental to atheroprotection. Its ability to promote cholesterol removal from peripheral tissues and facilitate its return to the liver for excretion positions apo A-1 as a crucial factor in maintaining lipid balance and preventing plaque formation in arteries [33, 34]. Furthermore, emerging research suggests that apo A-1’s anti-inflammatory and antioxidative properties may have therapeutic potential in treating conditions characterized by chronic inflammation and oxidative stress, which are often precursors to cardiovascular events. These multifaceted roles of apo A-1 underscore its significance beyond traditional lipid metabolism, highlighting its potential as a target for novel therapeutic strategies in cardiovascular disease prevention and management [34].

Apo A-1, as a predictive cardiovascular disease (CVD) risk biomarker, offers several clinical advantages. Unlike HDL-C and total cholesterol, apo A-1 can be evaluated using a simple test and does not require a fasting serum sample. HDL-C encompasses a diverse group of particles that vary in size, lipid and apolipoprotein composition, and density. During an acute-phase response, the conversion of HDL from an anti-inflammatory particle to a pro-inflammatory particle is characterized by modified protein configuration, amplified levels of ceruloplasmin and serum amyloid A (SAA), and reduced apo A-1 levels. This alteration of HDL composition is associated with increased HDL dysfunction. Furthermore, oxidative damage to HDL-C results in poor apo A-1 functionality in the arterial wall, which can diminish the competence of HDL-C/apo A-1 to promote cholesterol efflux from macrophages, thereby promoting CVD. Our findings suggest that preoperative high HDL-C/apo A-1 ratios, which signify cholesterol-rich HDL particles, increase CVD risk. In contrast, reduced postoperative HDL-C/apo A-1 ratios express improved HDL function. This improvement indicates better cholesterol efflux capacity and enhanced anti-inflammatory properties of HDL particles post-surgery. Incorporating apo A-1 measurement into routine practice could enhance the assessment and management of CVD risk in patients with obesity who are undergoing LSG [35–37].

By using apo A-1 as a marker, healthcare providers can better understand a patient’s cardiovascular health and the effectiveness of the surgical intervention. This could lead to more tailored and effective treatment plans, improving patient outcomes and potentially reducing the financial burden associated with cardiovascular complications. Additionally, these findings could encourage the inclusion of apo A-1 and HDL-C/apo A-1 ratios in clinical guidelines for managing cardiovascular risk in patients undergoing MBS. Such an approach could refine risk stratification and intervention strategies, ultimately contributing to better prevention and management of CVD in this population. Future research should focus on validating these findings in larger, multi-center studies and exploring the potential benefits of integrating these biomarkers into comprehensive cardiovascular risk assessment models.

Additionally, these findings could encourage the inclusion of apo A-1 and HDL-C/apo A-1 ratios in clinical guidelines for managing cardiovascular risk in bariatric patients. Such an approach could refine risk stratification and intervention strategies, ultimately contributing to better prevention and management of CVD in this population. Future research should focus on validating these findings in larger, multi-center studies and exploring the potential benefits of integrating these biomarkers into comprehensive cardiovascular risk assessment models. The observed postoperative reduction in MHR, while not correlating with hs-CRP in our study, presents an intriguing aspect of the complex interplay between inflammation and lipid metabolism post-MBS. This contrasts with Yayla et al.’s findings, where elevated MHR was linked to both increased hs-CRP and slower coronary blood flow [38]. This variance could reflect the multifaceted nature of MBS of MHR as a biomarker, which encapsulates both inflammatory status (monocyte levels) and lipid profile (HDL-C levels). Monocytes, as key players in inflammation, can have varying roles depending on the stage of recovery and the overall metabolic status of the patient post-MBS. Additionally, HDL-C, known for its role in reverse cholesterol transport and anti-inflammatory properties, might exhibit changes in functionality post-surgery that are not entirely captured by hs-CRP levels. This highlights the possibility that MHR could reflect more subtle shifts in the inflammatory and lipid metabolic landscape that hs-CRP alone might not fully reveal [7]. The MHR has emerged as a significant biomarker in cardiovascular health, particularly in coronary artery disease (CAD). A study demonstrated that elevated MHR levels are independently associated with increased all-cause, cardiovascular, and non-cardiovascular mortality in CAD patients, especially those with hypertension [37]. This suggests a higher inflammatory state and an increased risk of complications like infection and organ dysfunction in patients with elevated MHR​​​​. MHR’s role extends to atherosclerosis progression, where monocytes contribute to plaque formation and rupture, while HDL acts protectively against these processes. This dual role reflects the complexity of cardiovascular disease mechanisms and the potential of MHR as a comprehensive indicator of inflammatory and atherogenic status [39].

Additionally, assessing MHR with a broader range of inflammatory and metabolic markers could provide a more comprehensive picture of cardiovascular risk post-MBS. Our findings emphasize the need for a more detailed exploration of MHR’s role in the context of obesity and MBS. Future research should consider the dynamic interactions between different components of inflammation and lipid metabolism, potentially uncovering novel insights into cardiovascular risk assessment and patient management post-MBS.

Regarding TC, our study identified a significant correlation with hs-CRP. The postoperative reduction in cholesterol levels can be attributed to reduced gastric volume, decreased production of gastric lipase, diminished sterol absorption, and a likely decrease in the hydrolysis of TGs and free fatty acids, all contributing to a postoperative decline in total cholesterol, whereby two studies showed that the RYGB and one-anastomosis gastric bypass were superior in TC loss over time compared to LSG [40, 41]. Nevertheless, all MBS procedures had significant decreases in metabolic biomarkers compared to preoperatively. Consequently, an increased TC/HDL-C ratio can indicate an increased atherogenic risk when the lipid profile is within a desirable range. Both the TC/HDL-C and LDL-C/HDL-C ratios are considered sensitive and specific indices of CV risk, reflecting an increase in the atherogenic component in the numerator and a decrease in the anti-atherogenic component in the denominator [42].

Correspondingly, these ratios displayed significant positive associations with hs-CRP, which was superior to HDL-C alone. Additionally, the LDL-C/HDL-C ratio may reflect the primary apolipoprotein components of LDL-C and HDL-C, respectively. This underscores the vital role of apoprotein components in influencing vascular biology and regulating lipoprotein metabolism. Furthermore, evaluating apo A-1 concentration and HDL ratios provides deeper insights into the pathophysiology of dyslipidemia, potentially facilitating the improvement of therapeutic targets.

Consequently, it could be suggested that the measurement of apo A-1 and the HDL-C/Apo A-I ratio be added to the standard measures of HDL-C in patients with obesity undergoing LSG to evaluate postoperative cardiovascular risk improvement. Incorporating these biomarkers into clinical practice could lead to more personalized and accurate cardiovascular risk assessments, allowing for better-tailored interventions and follow-up strategies [35–37].

By highlighting the importance of these biomarkers, our findings could influence clinical guidelines by recommending their routine measurement in managing patients with obesity undergoing MBS. This could enhance the ability of healthcare providers to monitor and address cardiovascular risks more effectively, ultimately improving patient outcomes. Additionally, these insights encourage further research into developing new therapeutic approaches targeting apoprotein components and HDL ratios, paving the way for advancements in treating dyslipidemia and cardiovascular disease.

## Limitations

Firstly, the assessment of low-grade inflammation in this study was based solely on one classic inflammatory biomarker, hs-CRP. This choice was made because hs-CRP is a cost-effective biomarker with significant prognostic value across all levels of cholesterol and cardiovascular risk scores. Additionally, visceral adipose tissue promotes serum CRP levels through increased signaling, aided by elevated serum leptin and decreased adiponectin levels. However, hs-CRP can also be elevated in other conditions, such as autoimmune diseases like rheumatoid arthritis. Therefore, incorporating additional inflammatory cytokines, such as interleukin-1β (IL-1β), IL-6, monocyte chemoattractant protein-1 (MCP-1), TNF-α, caspase-3 (CASP-3), and platelet-derived growth factor subunit A (PDGF subunit A), in future studies could provide a more comprehensive understanding of the inflammatory profile and its modulation post-surgery.

Secondly, the study’s follow-up period was limited to 6 months post-surgery, capturing primarily short-term effects. Extending the follow-up duration beyond 12 and 18 months would be more beneficial for evaluating the long-term influence of inflammatory cytokines and ascertaining sustained improvements in apo A-1, lipid profile, and HDL-C ratios.

Thirdly, although our sample size of 78 patients was sufficient to detect medium effect sizes with increased power, a larger sample size could further enhance the validity of our findings. A bigger cohort would allow us to correct for more confounding factors, measure additional outcomes, and improve the generalizability of the results. Including participants from multiple centers in future research would also mitigate potential biases and enhance the robustness of the study’s conclusions.

Fourthly, no information was collected about the patients’ body composition, such as waist circumference and visceral fat, and their effects on inflammatory biomarkers. This additional information could provide more insights into where the patients lost their weight (extremities, visceral obesity, arms-legs-trunk), potentially enhancing the interpretation of the study results.

## Conclusion

This study highlights the effectiveness of the LSG in improving cardiovascular risk factors, particularly in managing inflammation, insulin resistance, and dyslipidemia, as evidenced by changes in HDL-C ratios and apo A-1 lipoprotein levels. Notable findings include a moderate association between the inflammatory marker hs-CRP and key cardiovascular biomarkers. However, further research is necessary to understand the broader implications of these findings in the management of obesity and to explore the potential of HDL-C/apo A-1 ratios in assessing HDL function post-LSG.

## Data Availability

Available with the corresponding author.

## Appendix. Full workup of all lab tastings

Fasting insulin levels were measured using ELISA (EIA-2935) [DRG International Inc., Springfield, NJ, USA]. Homeostasis model assessment of insulin resistance (HOMA-IR) was used to evaluate insulin resistance (fasting serum insulin (μIU/ml) × fasting serum glucose (mmol/l)/22.5).

Serum hs-CRP level was measured by a nephelometric assay (Behring Diagnostics, Marburg, Germany). Serum apo A-1 was determined by ELISA kit **(Cat no:** KE00157) [Proteintech Group, Inc., 5500 Pearl Street, Rosemont, IL, USA].

High-density lipoprotein risk ratios were calculated as follows: LDL-C/HDL-C was calculated by dividing LDL-C (mg/dl) by HDL-C (mg/dl); normal range should be < 3 in males, < 2.5 in females, TC /HDL-C by dividing TC (mg/dl) by HDL-C (mg/dl); normal range should be below < 4.5 in males, < 4 in females. Similarly, TG/ HDL-C ratio, normal range < 2 [21]. Monocyte was evaluated using Sysmex XN-2800 automated hematology analyzer (Japan) and MHR was calculated as the monocyte count (10^9^/µl)/HDL-C (mg/dl).
